# Validation of MELD 3.0 and ReMELD-Na scoring systems: a German clinical cohort study

**DOI:** 10.1007/s00423-025-03846-x

**Published:** 2025-09-25

**Authors:** Aghnia J. Putri, Decan Jiang, Zoltan Czigany, Arianeb Mehrabi, Markus Wolfgang Buechler, Uta Merle, Christoph Michalski, Peri Husen

**Affiliations:** 1 Department of General, Visceral, and Transplantation Surgery, University Hospital of Heidelberg, Heidelberg, Germany; 2 Department of Gastroenterology and Hepatology, University Hospital of Heidelberg, Heidelberg, Germany

**Keywords:** Liver transplantation, MELD, Scoring systems, Mortality, Equality

## Abstract

**Background:**

The Model for End-Stage Liver Disease (MELD) score, introduced in 2002, has since been refined. MELD 3.0, launched in 2023 in the United States, improves mortality prediction by incorporating sex and albumin. Since March 2025, Germany started to use reMELD-Na to improve prioritization of patients on the waiting list for liver transplantation (LT). This study compares the performance of original MELD, MELD-Na, MELD 3.0 with and without albumin, and reMELD-Na for patients waitlisted for LT in a large German transplant center.

**Methods:**

This retrospective single-center study included 206 listed patients from 2017 to 2021 for LT. Reclassification patterns along with predictive accuracy for three-month survival and overall survival (OS) of five different MELD scores were assessed using Harrell’s c-index and integrated area under the curve (iAUC).

**Results:**

Over a median follow-up of 33.9 months, 100 patients (51.5%) underwent LT, with a post-transplant survival rate of 70%. Thirty-eight patients (18.4%) received LT and sixteen patients died within the first three months after listing. ReMELD-Na and MELD 3.0 without albumin demonstrated the highest discrimination for three-month survival (c-index 0.848 and 0.827, respectively). Original MELD showed the poorest discrimination. MELD 3.0 without albumin showed the best overall performance in predicting OS (c-index 0.827) in males, while reMELD-Na performed best in females (c-index 0.705). Females with higher original MELD scores tended to receive even higher scores with MELD 3.0.

**Conclusion:**

This is the first German study to validate reMELD-Na and MELD 3.0, showing superior predictive performance over original MELD. MELD 3.0 may better reflect disease severity in women at advanced stages due to more upward reclassification.

## Introduction

The Model for End-Stage Liver Disease (MELD) score has revolutionized the liver transplantation (LT) allocation systems. Originally developed to predict mortality following transjugular intrahepatic portosystemic shunt (TIPSS) placement, it was later found to be a valuable tool for estimating mortality risk among patients listed for LT. The MELD score was officially introduced for LT purpose in 2002 and has undergone several updates since then [1, 2]. The second iteration of the MELD system in 2007, MELD-Na, improved prognostic accuracy by incorporating serum sodium levels [3]. In the United States, the most recent version of the MELD score, known as MELD 3.0, was implemented by United Network for Organ Sharing (UNOS) in 2023 [4]. By including new variables such as sex and serum albumin, MELD 3.0 provides a more nuanced assessment of mortality risk, that yielded to improvements in mortality prediction and transplant outcomes among LT recipients. This adjustment is particularly important given the systemic disadvantages women face at multiple stages of the transplant process from referral and evaluation to listing and ultimately receiving a LT [4]. Notably, steatotic liver disease (SLD) has emerged as the leading indication for LT among women without hepatocellular carcinoma, a trend expected to continue in the coming years. Despite its widespread adoption, regional differences in patient demographics, healthcare access, and outcomes underscore the need for local validation.

After relying on the original MELD score since its adoption in 2002, Germany has recently transitioned to using a refitted version of MELD-Na (reMELD-Na) as of March 2025 [5], following its implementation by Eurotransplant (ET). It was developed by reweighing the coefficients and re-establishing clinically meaningful lower and upper bounds for creatinine, bilirubin, INR, and sodium. Unlike the original MELD score, which capped a significant proportion of patient values and potentially overemphasized renal dysfunction, reMELD-Na limits such distortions by refining parameter bounds and reducing the disproportionate influence of creatinine [6]. Importantly, its calculation on sodium enhances its ability to identify high-risk patients with hyponatremia.

The present study seeks to address a gap in the existing literature by examining the performance of both MELD 3.0 and reMELD-Na at one of Germany’s largest LT centers, and situating these findings within the broader international developments in MELD-based scoring systems.

## Methods

### Study population

This study is a retrospective clinical cohort study designed to assess the validity of the MELD 3.0 [4] and reMELD-Na [6] scoring systems in predicting mortality and transplant outcomes among patients listed for LT at our center. Informed consent was obtained. The analysis is based on a dataset from the Department of General, Visceral and Transplant Surgery at the University Hospital of Heidelberg, Germany. The study included adults (≥ 18 years) listed for liver transplantation at our center between January 1, 2017, and December 31, 2021, with a minimum follow-up of three years. Patients with high-urgency (HU) status, re-transplantations, listings under standard exception criteria, or incomplete data were excluded. According to the Eurotransplant Liver Allocation System (ELAS), HU status is reserved for patients with an imminent risk of death, most commonly in the context of acute liver failure, acute graft failure, or other selected urgent conditions, as outlined in the Eurotransplant Manual (version 6.14, March 2025). Eligibility of HU is determined based on defined diagnostic and clinical criteria (e.g., King’s College or Clichy criteria) and requires confirmation through a formal audit process.

### Statistical analysis

Patient characteristics were reported as mean ± standard deviation for continuous variables and as frequencies (percentages) for categorical variables. Subgroup and sensitivity analyses were conducted based on sex (male vs. female). A two-sided p-value < 0.05 was considered statistically significant. The predictive accuracy of MELD models was assessed using Harrell’s c-index and Heagerty’s integrated time-dependent AUC (iAUC). Differences in c-index estimates with 95% confidence intervals were analyzed. All analyses were conducted in R Studio (Posit PBC version 4.4.3) and in consultation with the Institute for Medical Biometry and Informatics, University of Heidelberg. The study was conducted in accordance with the latest version of the Declaration of Helsinki and the Declaration of Istanbul. Ethical approval was obtained from the local ethics committee (S-105/2025).

## Results

### Demographic and clinical characteristics

During the study period from 2017 to 2021, a total of 392 patients were listed for LT. The mean age of the whole cohort was 53.19 ± 10.91 years, with 62.5% of the patients being male. Patients listed for high urgency transplantation (*n* = 42 patients), re-transplantation (*n* = 51 patients), or with malignancies qualifying for standard exception criteria (e.g., HCC, neuroendocrine tumors) (*n* = 87 patients) were excluded. In addition, six patients were excluded due to incomplete data resulting from transfer to another center. Upon exclusion, the final study population consisted of 206 patients, with a mean age of 53.08 ± 10.35 years and a male proportion of 59.2% (Table 1). Within three months of listing, 22 patients were removed from the waiting list due to worsening clinical condition, and nine patients due to improvement.

**Table 1:** Patient Characteristics

Variables	Sex		All	*p*
	M (*n* = 122)	F (*n* = 84)		
Age (Mean, SD) Age (Median, IQR)	52.02 ± 10.14 54 (48–59)	54.62 ± 10.51 58 (52–61)	53.08 ± 10.35 55 (49–60)	0.08
Etiology (n, %)				0.25
Alcohol	51 (56.7)	39 (43.3)	90 (43.7)	
PSC	20 (66.7)	10 (33.3)	30 (14.6)	
MASLD	6 (42.8)	8 (57.2)	14 (6.8)	
Chronic HBV/HDV	7 (53.8)	6 (46.2)	13 (6.3)	
PBC	9 (75)	3 (25)	12 (5.8)	
Hepatitis C	8 (88.8)	1 (11.2)	9 (4.4)	
Autoimmune hepatitis	2 (33.3)	4 (66.7)	6 (2.9)	
SSC	3 (100)	0 (0)	3 (1.5)	
Cryptogenic	4 (40)	6 (60)	10 (4.9)	
Others	12 (63.1)	7 (36.9)	19 (9.2)	
MELD score at listing				
Original MELD	18.25 ± 8.02	17.32 ± 8.52	17.87 ± 8.22	0.42
MELD-Na	20.02 ± 8.49	18.82 ± 8.69	19.53 ± 8.57	0.32
MELD 3.0 without albumin	20.34 ± 8.58	19.10 ± 8.78	19.83 ± 8.67	0.31
MELD 3.0 with albumin	20.68 ± 8.80	19.54 ± 8.57	20.21 ± 8.87	0.36
ReMELD-Na	16.78 ± 7.15	15.80 ± 7.20	16.38 ± 7.17	0.41

*HBV* hepatitis B virus, *HDV* hepatitis D virus, *IQR* interquartile range, *MELD* Model for End-Stage Liver Disease, *Na* sodium, *MASLD* metabolic dysfunction-associated steatotic liver disease, *PSC* primary sclerosing cholangitis, *SD* standard deviation, *SSC* secondary sclerosing cholangitis.

Alcohol-related cirrhosis was identified as the most common etiology of liver disease, accounting for 90 cases (43.7%), followed by primary sclerosing cholangitis (PSC), which was observed in 30 cases (14.6%). Metabolic dysfunction-associated steatotic liver disease (MASLD) was present in 14 patients (6.8%), of whom 57% were female. During the study period, 100 patients eventually underwent LT, leaving 106 patients untransplanted until the end of the observation period. MELD scores of the study cohort were calculated using various scoring systems. The original MELD score was 17.8 ± 8.22, MELD-Na was 19.53 ± 8.57, MELD 3.0 without albumin was 19.83 ± 8.67, MELD 3.0 with albumin was 20.21 ± 8.87, and ReMELD-Na was 16.38 ± 7.17. Across all MELD formulas, female patients exhibited lower scores.

Age and etiology of liver disease showed no statistically significant association with either short-term or long-term survival in non-transplanted patients using the original MELD formula. In relation to sex, female patients have a 23% lower relative risk of survival during the observed period. However, this difference did not reach statistical significance (Table 2). For overall survival, the hazard ratio for female patients was 0.96, suggesting comparable long-term outcomes between sexes (*p* = 0.88) (Table 2).

**Table 2:** Correlation of patient characteristics with Three-Month survival and overall survival in Non-Transplanted patients within Three-Months of Listing, (Listed using the original MELD Score)

		Three-month survival			Overall survival			
	All *n* = 168	Male *n* = 99	Female *n* = 69	*P*	All *n* = 168	Male *n* = 99	Female *n* = 69	*P*
Age				0.18				0.12
Etiology				0.69				0.71
Sex	151 (89.8)	88 (88.8) ref.	63 (91.3) **HR 0.77**	0.62	98 (58.3)	58 (58.5) ref.	49 (71) **HR 0.96**	0.88

### Reclassification of Waitlisted Patients using Different MELD Scores

Reclassification across MELD scoring systems was performed to evaluate the pattern of score shifts resulting from the application of different calculation formulas. For this analysis, MELD Original, MELD-Na, and MELD 3.0 were included. ReMELD-Na was excluded due to its capped maximum value of 36, which hindered a direct one-to-one comparison with the other scoring systems. Reclassification of LT candidates was assessed by comparing categorical thresholds across the scoring systems: <10, 11–19, 20–29, 30–39, and ≥ 40. A contingency table was constructed to evaluate the extent of changes in patient categorization following the application of MELD 3.0, both with and without albumin. When MELD 3.0 was applied, notable reclassifications were observed, with some patients moving to higher and others to lower MELD categories. Upward reclassification occurred more frequently in male patients within the lower MELD categories, indicating a shift in perceived severity under the updated model. Conversely, among patients with higher original MELD scores, female patients were more likely to receive even higher MELD 3.0 scores, suggesting that MELD 3.0 may more accurately capture disease severity in women at advanced stages of liver disease in comparison to original MELD (Table 3)

**Table 3:** Reclassification of patients following MELD score adjustment (A. MELD original vs. MELD 3.0; B. MELD-Na vs. MELD 3.0)

A.	MELD 3.0 without Albumin				
MELD Original	< 10	10–19	20–29	30–39	40
< 10	19 (M: F, 11:8)	8 (M: F, 5:3)	-	-	-
10–19	-	82 (M: F, 44:38)	20 (M: F, 11:9)	-	-
20–29	-	4 (M: F, 2:2)	46 (M: F, 32:14)	8 (M: F, 6:2)	-
30–39	-	-	-	6 (M: F, 5:1)	6 (M: F, 2:4)
40	-	-	-	-	7 (M: F, 4:3)
	** MELD 3.0 with Albumin**				
MELD Original	**< 10**	**10–19**	**20–29**	**30–39**	**40**
< 10	19 (M: F, 11:8)	8 (M: F, 5:3)	-	-	-
10–19	1 (M: F, 1:0)	77 (M: F, 42:35)	24 (M: F, 12:12)	-	-
20–29	-	-	47 (M: F, 32:15)	11 (M: F, 8:3)	-
30–39	-	-	-	3 (M: F, 3:0)	9 (M: F, 4:5)
40	-	-	-	-	7 (M: F, 4:3)
B.	** MELD 3.0 without Albumin**				
MELD-Na	**< 10**	**10–19**	**20–29**	**30–39**	**40**
< 10	18 (M: F, 11:7)	5 (M: F, 2:3)	-	-	-
10–19	1 (M: F, 0:1)	83 (M: F, 46:37)	6 (M: F, 3:3)	-	-
20–29	-	6 (M: F, 3:3)	58 (M: F, 39:19)	5 (M: F, 4:1)	-
30–39	-	-	2 (M: F, 1:1)	9 (M: F, 7:2)	5 (M: F, 1:4)
40	-	-	-	-	8 (M: F, 5:3)
	** MELD 3.0 with Albumin**				
MELD Na	**< 10**	**10–19**	**20–29**	**30–39**	**40**
< 10	18 (M: F, 11:7)	5 (M: F, 2:3)	-	0	-
10–19	2 (M: F, 1:1)	78 (M: F, 44:34)	10 (M: F, 4:6)	0	-
20–29	-	2 (M: F, 1:1)	59 (M: F, 39:20)	8 (M: F, 6:2)	-
30–39	-	-	2 (M: F, 1:1)	6 (M: F, 5:1)	8 (M: F, 3:5)
40	-	-	-	-	8 (M: F, 5:3)

*MELD* The Model for End-Stage Liver Disease, *Na* Sodium, *F* Female, *M* Male

Among 19 patients initially classified with an original MELD score of < 10, eight were reclassified into the 11–19 category under MELD 3.0 without albumin, while 19 remained in the same category. In the 11–19 group, the majority (82 patients) retained their classification, but 20 patients were reclassified into the 20–29 range, with a nearly equal distribution between male and female patients. In the original 20–29 category, 46 patients remained unchanged, whereas eight were reclassified into the higher-risk 30–39 category and four were downgraded to the 11–19 range. Among those originally scored in the 30–39 group, six remained in the same category, while another six were reclassified into the 40 group, two-thirds of whom were female. All patients originally categorized as MELD 40 remained in that category, indicating no downgrades among the highest-risk patients. When comparing MELD-Na to MELD 3.0 without albumin, most patients remained in their original risk category. However, reclassifications also occurred, but not as much in comparison to the original MELD. Among those initially scored < 10 by MELD-Na, five patients were reclassified into the 10–19 group under MELD 3.0. Within the 10–19 group, 83 patients remained stable, but seven patients were reclassified; six into the 20–29 category and one downgraded to < 10. Reclassifications into higher categories continued among MELD-Na 20–29 patients, where five patients shifted to either 30–39, where four of them were male. Among patients in the 30–39 category, seven were reclassified, which two moved downward to 20–29 and five moved upward into the ≥ 40 category, with four of the five being female. All eight patients originally categorized as MELD 40 retained their classification under MELD 3.0. A similar trend was observed when comparing MELD-Na to MELD 3.0 with albumin. Although fewer patients were reclassified compared to the version without albumin, upward shifts were still evident, particularly in female patients within higher MELD categories. For instance, of the eight patients moved into the MELD ≥ 40 category from 30–39, five were female. Overall, the reclassification trends show that MELD 3.0, particularly the version without albumin, more frequently increases MELD scores for women with advanced liver disease, whereas men were more commonly upshifted from lower MELD categories

### Predictive performance and discriminative ability of different MELD scores

During the median observational period of 33.90 months (IQR 11.55–59.38), 100 patients (51.5%) underwent LT, with a post-transplant survival rate of 70% at the end of the observation period. Of the 106 patients who did not undergo LT until the end of the study period, 54 patients (50.9%) remained alive by the end of the study. Thirty-eight patients (18.4%) received their transplant within three months of being listed according to original MELD score. Sixteen patients died within three months of being listed. The most common causes of death were liver failure (*n* = 7) and sepsis (*n* = 7), followed by bleeding, primarily due to fundic variceal hemorrhage. The predictive performance of MELD-based scoring systems was evaluated for both three-month and overall survival. Discriminative ability was assessed using Harrell’s concordance index (c-index) and the integrated area under the curve (iAUC) (Table 4). The analysis includes only patients who were still not transplanted within three months of listing, resulting in a cohort of 168 patients.

**Table 4:** Comparative discrimination ability of MELD original and MELD 3.0

MELD Scores	Three-month survival			Overall survival		
	Harrel c-index			Harrel c-index		
	All	M	W	All	M	W
MELD Original	0.794	0.861	0.744	0.794	0.860	0.688
MELD-Na	0.824	0.908	0.702	0.824	0.907	0.680
MELD 3.0 without Albumin	0.827	0.917	0.709	0.827	0.917	0.682
MELD 3.0 with Albumin	0.809	0.885	0.712	0.809	0.884	0.686
ReMELD-Na	0.848	0.897	0.755	0.820	0.897	0.705

*c-index* Harrell’s concordance index, *MELD* The Model for End-Stage Liver Disease, *Na* Sodium

In our cohort, reMELD-Na achieved the highest predictive accuracy for three-month survival, with a c-index of 0.848 in the overall population. This was followed closely by MELD 3.0 without albumin (c-index 0.827) and MELD-Na (c-index 0.824). MELD 3.0 with albumin showed slightly better accuracy (c-index 0.809) compared with the original MELD (c-index 0.794).

**Fig. 1:**
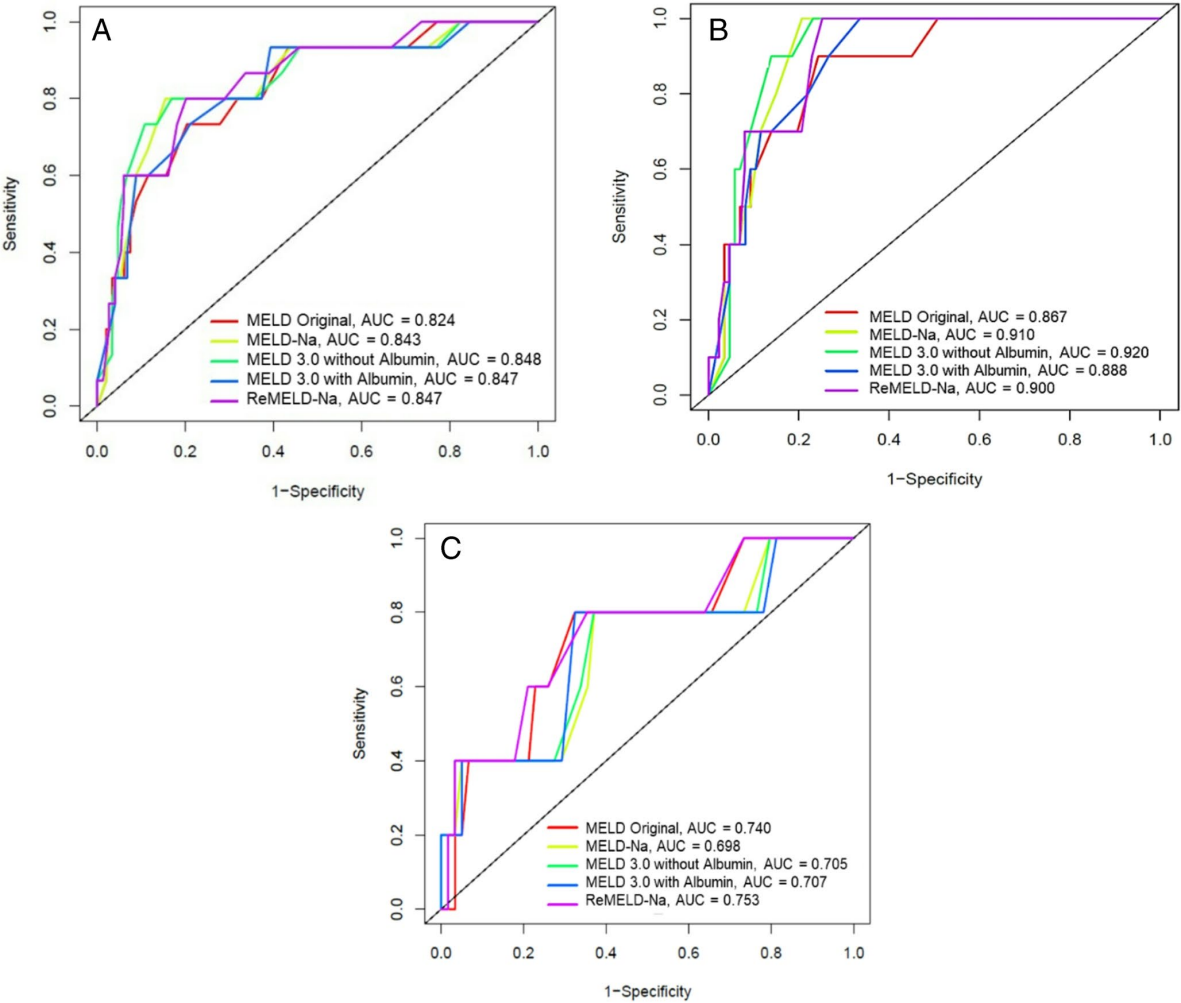
Predictive Value of MELD Scores for 3-Month Survival in Waitlisted Patients in (**A**) All Patients, (**B**) Men and (**C**) Women. MELD 3.0 without albumin achieved the highest AUC for three-month survival (0.848), followed closely by reMELD-Na and MELD 3.0 with albumin (both 0.847) and MELD-Na (0.843), while MELD Original had the lowest AUC (0.824). Among men, MELD 3.0 without albumin was superior (AUC 0.920), followed by MELD-Na (0.910) and reMELD-Na (0.900). Among women, reMELD-Na outperformed all other scores (AUC 0.753).

When stratified by sex (Fig. 1), MELD 3.0 without albumin yielded the strongest performance among male patients, with c-index of 0.917 and AUC of 0.920, followed by MELD-Na (c-index 0.908, AUC 0.910) and reMELD-Na (c-index 0.902, AUC 0.900). Female patients demonstrated generally lower model performance, consistent with existing literature on sex-based disparities in MELD-based scores. Nevertheless, reMELD-Na provided the best three-month survival prediction in women, with c-index of 0.755 and AUC of 0.753, followed by MELD Original (AUC 0.740, c-index 0.744). MELD 3.0 with albumin (c-index 0.712, AUC 0.707), MELD 3.0 without albumin (c-index 0.709, AUC 0.705), and MELD-Na (c-index 0.702, AUC 0.698) showed lower performance.

**Fig. 2:**
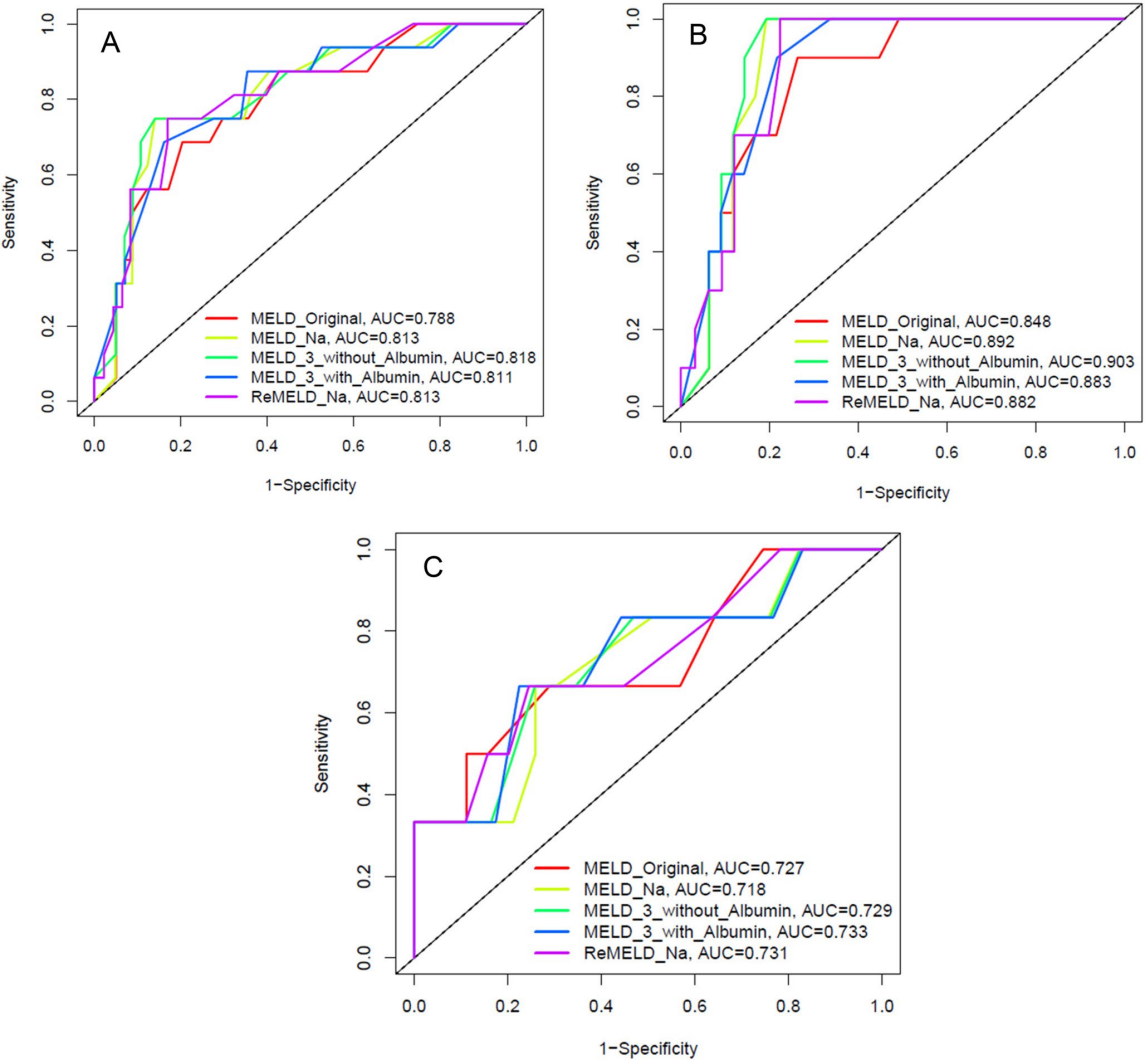
Predictive Value of MELD Scores for Overall Survival in Waitlisted Patients in (**A**) All Patients, (**B**) Men and (**C**) Women. For overall survival, MELD 3.0 without albumin ranked highest (AUC 0.827), followed by MELD-Na (0.824), reMELD-Na (0.819), and MELD Original (0.794). By sex, MELD 3.0 without albumin was best in men (AUC 0.922), while reMELD-Na led in women (AUC 0.707)

For overall survival (Figure 2), MELD 3.0 without albumin maintained strong predictive led performance in the overall cohort, with both an AUC and c-index of 0.827. ReMELD-Na and MELD-Na each followed with c-indices of 0.820 and 0.824 (AUC 0.819 and 0.824), respectively. MELD Original remained the least accurate (c-index 0.794, AUC 0.794). Among men, MELD 3.0 without albumin was superior (c-index 0.827, AUC 0.922), followed by MELD-Na (c-index 0.824, AUC 0.912) and reMELD-Na (c-index 0.897, AUC 0.902). In women, reMELD-Na yielded the highest discriminative performance (c-index 0.705, AUC 0.707), however, slightly ahead of MELD 3.0 with albumin (c-index 0.686, AUC 0.698), MELD 3.0 without albumin (c-index 0.682, AUC 0.698), MELD-Na (c-index 0.702, AUC 0.718), and MELD Original (c-index 0.688, AUC 0.727)

## Discussion

These findings suggest that MELD 3.0 (particularly without albumin) and reMELD-Na provide improved predictive accuracy for short-term and long-term survival in LT candidates in the study cohort, outperforming MELD-Na and original MELD. Notably, MELD 3.0 without albumin yielded the highest predictive accuracy in men, while reMELD-Na comparatively performed best in female patients and followed by MELD 3.0 without albumin. This finding aligns with the known limitations of prior MELD-based models in accurately estimating mortality risk in women [4, 7]. While the German and American transplant populations differ slightly, particularly in terms of racial diversity, the underlying disease profiles and allocation priorities are largely comparable [8–10]. Sex-based differences in liver disease and transplant allocation have been previously reported and appear relevant in our cohort. Female patients are disproportionately affected by conditions such as MASLD (metabolic dysfunction-associated steatotic liver disease), while male patients more commonly present with alcohol-related cirrhosis [11, 12]. Additionally, women have historically had lower access to transplantation, even in systems with shorter waiting times, due to factors including smaller body size, lower creatinine levels, hence affecting the proportionate of the MELD score, and potential implicit bias [13]. The mean original MELD of this cohort is 17.87 ± 8.22, which is slightly higher than the trend in Europe and South Korea, but comparable to the United States [7, 14, 15]. The inclusion of sex as a variable is critical, as previous MELD versions consistently underestimated disease severity in women due to their lower serum creatinine levels, which often fail to reflect true renal impairment [4, 15]. By explicitly adjusting for female sex and incorporating albumin, MELD 3.0 provides a more adequate risk profile for LT candidates, particularly those disadvantaged by prior models [4, 7, 16]. Compared to the U.S. cohort, in which MELD 3.0 achieved a c-index of 0.869, our cohort demonstrated a c-index of 0.809 for both three-month and overall survival. This is still relatively comparable and higher than that reported in a South Korean cohort, where the c-index was 0.564 [4, 7]. MELD 3.0 also reclassified a greater number of female patients to higher priority categories, thereby addressing a long-standing gender inequity in waitlist mortality, which has been shown in several cohorts [4, 7, 16]. This enhancement addresses one of the critical limitations of earlier MELD versions: the underestimation of disease severity in women, partly due to the reliance on serum creatinine. Thus, it was expected and confirmed in our study that MELD 3.0 improved predictive performance. On the other side, although not incorporating gender specifically in the formula, reMELD-Na focuses on optimizing the existing variables, which incorporates bilirubin, creatinine, INR, and sodium, by reweighing their coefficients and redefining their upper and lower bounds based on data-driven thresholds according to European database [5, 6, 17]. While reMELD-Na does not explicitly include sex or albumin, its parameter recalibration indirectly benefits female patients by down-weighting creatinine and optimizing the ratio of sodium in the formula [6]. In our study, these advantages were reflected in the comparative performance metrics in female cohort.

This study confirmed that both MELD 3.0 and reMELD-Na outperform older scoring systems, offering enhanced prognostic accuracy and improved fairness. MELD 3.0 addresses systemic sex disparities by explicitly modeling female sex and hypoalbuminemia, while reMELD-Na achieves equitable performance through rigorous regional recalibration and parameter optimization. The strong performance of reMELD-Na in women, despite the absence of a sex term, also highlights the value of data-driven model refitting in improving transplant equity across diverse patient subgroups. Compared to MELD-Na, reMELD-Na more accurately reflects true mortality risk by using updated coefficients and narrower parameter caps, leading to a better fit for European patients [6, 17, 18]. The reclassification trends in our study show that MELD 3.0, particularly the version without albumin, more frequently increases MELD scores for women with advanced liver disease, whereas men were more commonly upshifted from lower MELD categories (MELD < 20). For example, women with original MELD scores in the 30–39 range often gained additional points under MELD 3.0, which should have improved their prioritization. These results suggest that MELD 3.0 may better reflect sex-specific differences in disease severity, which has also been shown in several studies [4, 7]. The pattern of upward reclassification was also more evident among non-HCC patients, with 27.6% experiencing a shift to a higher MELD category, compared to 12.5% among patients with HCC [19]. 

Due to its retrospective nature, MELD’s use of serum albumin and sodium in this study may be confounded by undocumented exogenous administration. Both models lack dynamic inputs that reflect rapid clinical deterioration or systemic inflammation [7]. Furthermore, as both scores primarily capture hepatic and renal dysfunction, it could fail to account for extrahepatic organ failures, such as respiratory and circulatory problem or refractory ascites, which limits their prognostic value in patients with acute-on-chronic liver failure (ACLF). MELD-based models have been shown to underestimate mortality risk in ACLF, especially in patients requiring mechanical ventilation or vasopressor support. These limitations highlight the gap between current MELD-based allocation and the complex, multi-organ nature of ACLF, and suggest that incorporating dynamic, multi-system parameters into prognostic models could better inform listing decisions for this high-risk population [20, 21].

 As one of the largest LT centers in Germany [8], the findings likely reflect broader national trends. It is, however, important to recognize that MELD-based models, by design, do not incorporate disease etiology or predict post-transplant outcomes, but rather estimate pre-transplant mortality risk. Prospective multicenter validation studies across the ET region are needed to determine whether reMELD-Na continues to improve equity and outcomes in diverse patient populations. Such studies should specifically assess whether sex-based disparities persist under reMELD-Na, and whether further refinements, such as incorporating a female-specific adjustment, could enhance equality in allocation.

Both MELD 3.0 and reMELD-Na represent significant advancements in mortality prediction for liver transplant candidates. Their implementation, in comparison to the previous MELD models, can enhance organ allocation strategies and potentially reduce disparities, particularly for female patients.

## Data Availability

No datasets were generated or analysed during the current study.

## Abbreviations

*ACLF*: acute-on-chronic liver failure
*ET*: Eurotransplant
*ELAS*: Eurotransplant Liver Allocation System
*HBV*: hepatitis B virus
*HDV*: hepatitis D virus
*HU*: high urgency
*IAUC*: integrated area under the curve
*IQR*: interquartile range
*LT*: liver transplantation
*MELD*: Model for End-Stage Liver Disease
*Na*: sodium
*PSC*: primary sclerosing cholangitis
*ReMELD-Na*: Refitted Model for End-Stage Liver Disease–Natrium -Score
*SD*: standard deviation
*MASLD*: metabolic dysfunction-associated steatotic liver disease
*SSC*: secondary sclerosing cholangitis
*TIPSS*: transjugular intrahepatic portosystemic shunt
*UNOS*: United Network for Organ Sharing
